# The Path to UVCB Ecological Risk Assessment: Grappling with Substance Characterization

**DOI:** 10.1002/etc.5462

**Published:** 2022-09-30

**Authors:** Daniel Salvito, Marc Fernandez, Jeremy S. Arey, Delina Y. Lyon, Nelson Lawson, Sandrine Deglin, Matthew MacLeod

**Affiliations:** ^1^ Retired; ^2^ Environment and Climate Change Canada Vancouver British Columbia Canada; ^3^ Oleolytics LLC, State College PA USA; ^4^ Pine Chemicals Association International Fernandina Beach Florida USA; ^5^ Department of Environmental Science and Analytical Chemistry Stockholm University Stockholm Sweden USA; ^6^ ExxonMobil Biomedical Science Annandale New Jersey USA; ^7^ Health and Environmental Sciences Institute Washington District of Columbia USA

**Keywords:** Complex mixtures, ecological risk assessment, mixtures, risk assessment, UVCB

## Abstract

Substances of unknown or variable composition, complex reaction products, and biological materials (UVCBs) pose a unique challenge to regulators and to product registrants, who are required to characterize their fate, exposure, hazard, and potential risks to human health and the environment. To address these challenges and ensure an efficient and fit‐for‐purpose process, it is proposed that the ecological risks of UVCBs be assessed following a tiered strategy. The development of this approach required exploring how substance composition ties into hazard and exposure information and determining the extent to which a UVCB needs to be characterized to ensure a robust risk assessment. The present study highlights the key aspects of this new method. It presents how a tiered substance characterization approach can be integrated into broader UVCB risk‐assessment schemes to encourage an examination of data needs before a full substance characterization is performed. The first tier of the characterization process, Tier 0, is a fundamental step that includes data from basic, lower‐resolution compositional analyses. Tier 0 assessments can be used to inform hazard and exposure for any substance of interest. The need for more sophisticated, higher‐tier characterization is determined by the level of uncertainty of the risk assessment. The next step will integrate a tiered exposure assessment into the characterization scheme featured in the present study, to create a more complete risk‐assessment framework. *Environ Toxicol Chem* 2022;41:2649–2657. © 2022 Her Majesty the Queen in Right of Canada, Health and Environmental Sciences Institute and The Authors. *Environmental Toxicology and Chemistry* published by Wiley Periodicals LLC on behalf of SETAC. Reproduced with the permission of the Minister of Environment and Climate Change Canada.

## INTRODUCTION

The compositional variability and the unknown or poorly characterized constituents of substances of unknown or variable composition, complex reaction products, or biological materials (UVCBs) pose unique risk‐assessment challenges for product registrants, regulators, and research scientists. Petroleum products, resins/rosins, and fragrances or other natural complex substances are good examples of UVCBs and can contain many constituents. Some of these constituents are partially unknown or variable in their chemistry and/or concentration, depending on fluctuations in their source material and their manufacturing process. (1) How does one measure or estimate exposure when constituents may have different chemical structures and physicochemical properties, and therefore different fates and toxicities? (2) Are our current models and test systems for single‐component organic compounds up to the task? (3) How does one obtain and interpret hazard data when a substance's composition may vary from batch to batch or when testing the whole substance may overlook a single constituent driving hazard or provide insufficient insight into the complex dissolution behavior of individual substance constituents (Salvito et al., 2020)?

Substance characterization is often a roadblock to further steps in the risk‐assessment process. Risk assessors may struggle with the overall complexity of a UVCB. (1) How much substance information is needed to confidently draw a conclusion on exposure and effects? (2) Are there less onerous steps that can be taken rather than developing a full and complete identification of every compound in substances that may contain hundreds or thousands of organic constituents? (3) When is a more detailed analysis required for a robust risk characterization that is fit for purpose?

There are computational challenges inherent in representing substances that are complex because they have multiple components with structural variability as well as physical–chemical properties that are predicted or measured across large ranges that cover multiple orders of magnitude (Dimitrov et al., 2015). Although online access to large chemical property databases and environmental risk profiling tools is increasing rapidly for hundreds of thousands of substances, their data may misrepresent UVCBs based on incorrect or overly simplified substance characterization. Such mischaracterization could result from the selection of a few constituents, based on undocumented criteria, to represent a UVCB of hundreds or thousands of constituents, for the purpose of modeling.

As part of the Health and Environmental Sciences Institute's (HESI's) Project on UVCB Ecological Risk Assessment, a workshop was convened in 2018 to address how substance composition links to hazard and exposure and what level of detail is needed to perform a risk assessment on UVCBs. In the present study, we report the key findings of the workshop and present a tiered scheme for substance identification that should prove useful in UVCB ecological risk assessment.

The output of this workshop provides a roadmap for a Tier 0 characterization of these complex substances. This Tier 0 assessment includes basic inventory information generally available for all chemicals used or manufactured in a country or jurisdiction. The fundamental problem in assessing these substances often lies in the endless loop of discussion that takes place when people try to create high‐quality substance characterizations. Such characterizations are often neither possible nor practical.

The workshop participants (scientists from the regulatory, academic, and business sectors) developed a methodology, which uses widely available information that can provide sufficient substance characterization to proceed with hazard and exposure assessment. Sometimes additional analytical effort may be required. Although Tier 0 information is meant to apply generically across multiple industrial sectors, higher‐tier characterizations will probably be more specific and have different requirements for different industrial sectors. The driving question discussed at the workshop was whether there was sufficient understanding of a substance's characterization, and a sufficiently low uncertainty of that characterization, to proceed with exposure and hazard assessment.

## REGULATORY CHARACTERIZATION GUIDANCE AND REQUIREMENTS

Accurately and comprehensively assessing the ecological and human health risks of a UVCB substance requires sufficient substance identification such that any deficiencies in the available whole‐substance information may be estimated using modeled data for individual constituents or representative chemical constituents (based on the substance identity information). Nomenclature approaches, such as the Chemical Abstracts Service registry number (CAS RN), are limited in an unambiguous identification of UVCBs (Salvito et al., 2020).

Substance identity and characterization requirements can be found in various guidance documents by the European Chemicals Agency (ECHA), and the Organisation for Economic Co‐operation and Development (OECD). Guidance on the identification and naming of substances can be found in ECCC (2005); ECHA (2014a) and OECD (2014a, 2015). Examples of guidance on the grouping of substances and the use of read‐across approaches can be found in OECD (2014b) and ECHA (2017b). Endpoint‐specific guidance can be found in ECHA (2008, 2014b, 2016, 2017c) and OECD (2018), and guidance pertaining to ecological assessment approaches can be found in ECHA (2017a).

Identity and characterization issues for UVCBs have also been considered more broadly in technical reports and articles available on mixtures in the environment (Adolfsson‐Eric et al., 2012; Backhaus & Faust, 2012; Dimitrov et al., 2015; European Centre for Ecotoxicology and Toxicology of Chemicals, 2011; Meek et al., 2011; Rasmussen et al., 1999). One of the more advanced approaches to UVCB characterization is hydrocarbon block methodology (HBM), which characterizes petroleum UVCBs based on “blocks” or groups of hydrocarbon components with similar physical–chemical, fate, and toxicity properties. Each block is then assigned a representative value for each property (CONCAWE, 1996). An example of this method and its associated risk‐assessment tools is PetroTox (CONCAWE, n.d.), which is a spreadsheet that calculates the toxicity of petroleum products to aquatic organisms. The HBM approach is the result of 30 years of work led by the petroleum sector.

Given the large number of constituents that may be present in UVCBs and their diversity and variability, it is often difficult to be sure of what data are pertinent for any particular UVCB undergoing ecological and/or human health risk assessment. This makes a case‐by‐case approach necessary for many UVCB evaluations. As technology advances and new ways to test a UVCB's hazard or fate properties used in risk assessment emerge, one should not preclude examining the more recent state of the science for additional suggestions in devising more specific testing requirements and assessment approaches for UVCBs.

## TYPES OF UVCBs

These substances have a wide range of functions, chemical structures, and properties and are produced by and used in many different sectors of industry. Consequently, they can be associated with different risk‐assessment challenges, including (1) level of complexity, variation, and uncertainty in composition; (2) poorly water‐soluble components; (3) ionogenic components (i.e., substances existing for a large part as charged species in environmental conditions); (4) surfactant properties; (5) single or multiple modes of action; and (6) unresolved components that cannot be separated analytically. Categorizing substances according to chemistry, production volumes, substance complexity, or other well‐chosen criteria could help determine how to prioritize characterizations.

This concept was illustrated using UVCBs from Environmental and Climate Change Canada's (ECCC) Domestic Substance List (2019). Four hundred nonpetroleum organic UVCBs were screened out of approximately 4000 existing substances that met the criteria for further regulatory action, including (1) inherent toxicity to human health or the environment that is persistent and/or bioaccumulative, and (2) substances with the greatest potential for exposure from the human health perspective. This subset of 400 substances was organized by chemical structure and/or functional class. Table 1 provides a snapshot of the number of substances (kilograms per year) in some of the main UVCB classes at a given point in time. Although this cursory analysis does not provide a rigorous and comprehensive inventory of the different classes of UVCBs, it does provide valuable information on the range and variety of chemical classes and their associated industrial sectors in the UVCB landscape.

**Table 1:** Structure and/or functional class mapping of substances of unknown or variable composition, complex reaction products, and biological material registered for the Canadian market

Chemical class name	Number of substances
Terpenes and terpenoids	67
Quaternary ammonium compounds	27
Resins and rosins	27
Fatty acids and salts	26
Aliphatic amines	25
Alkylbenzene sulfonic acids and derivatives	24
Pigments and dyes	23
Individual substances	22
Naphthenic acids and their salts	15
Fatty amides	14
Chlorinated paraffins	11
Dithiophosphate alkyl esters	11
Phosphoric acid derivatives	11
Siloxane and silanes	10
Substituted diphenylamines	9
Fatty esters and derivatives	8
Hindered phenols	6
Perfluoro substances	6
Anthraquinones	5
Nitriles	5
Nonylphenols and derivatives	5
Proteins and derivatives	5

Environment and Climate Change Canada, Domestic Substance List (2019).

The workshop attendees concluded that UVCBs could be grouped into three major categories: (1) products of chemical reactions, (2) products extracted/purified from sources in nature, and (3) reaction products of UVCBs.

Products of chemical reactions were discussed using the manufacturing process of certain surfactants as examples. In these examples, the composition of the reactants was known, and the reaction product (including reactant residues) was either predictable or easily measurable analytically, so it was possible to define substance composition for hazard and exposure assessments.

Products extracted/purified from sources in nature represent the largest tonnage fraction of all UVCBs. These include petroleum substances (Supporting Information, S1) or smaller‐volume commercial materials, such as essential oils used in fragrances. Although detailed analytical information is frequently available for some of these substances, the precise chemical structures of many of their constituents remain unidentified because of analytical limitations, especially for constituents present in low concentrations. For example, current analytical approaches may be unable to distinguish between closely related constituents that coelute, may require multiple analytical techniques to capture all constituents, or may have insufficient detection limits to determine the individual concentrations of potentially hundreds or thousands of constituents, depending on the UVCB under analysis. Variations in the source material compounds these difficulties, but chemical structures can sometimes be inferred based on the source of the parent material and the extraction methodology. For example, depending on the extraction method, citrus extracts would be expected to contain limonene. This concept is further discussed in Supporting Information, S1.

The attendees also addressed the reaction products of UVCBs which, in contrast to monoconstituent substances, present an increased level of challenge in compositional identification. The example discussed at the workshop was the glycerol ester of rosin (Supporting Information, S2). This substance is a chemically treated (acetylated) resin extract from pine and other trees and consists of various terpenes. There is often good analytical understanding of the source material of this type of substance, making the chemistry of reaction products predictable (Supporting Information, S2).

## UNCERTAINTY AND/OR VARIABILITY IN COMPOSITION FOR SUBSTANCE IDENTIFICATION

The “U” in UVCB refers to unknowns in the identity and potentially the proportion of substance constituents. The CAS RN and basic nomenclature information with limited compositional or manufacturing process details are available for thousands of existing substances that have been nominated or registered in chemical inventories.

The unknown fraction of a UVCB can be composed of constituents that are not characterized but can be deduced from the structure of the starting material and process information (“known unknowns”) but can also be composed of constituents for which no structural information can be accessed and that require thorough substance characterization (“unknown unknowns”). The unknown fraction of a UVCB may also contain groups of structurally related but distinct constituents that are difficult or impossible to resolve analytically and are present in low concentrations. Inevitably, the unknown fraction of UVCB substance constituents leaves most assessors with limited information with which to make accurate prioritizations or risk assessments of these substances.

The variability in UVCBs stems from the source material and the production process. The process by which a UVCB is produced is typically tightly controlled and includes quality checks to ensure product consistency and adherence to technical specifications. However, slight changes or variations in the process can still result in compositional variability without affecting the function of the substance. Furthermore, because many UVCBs are produced or extracted from natural substances, variations in the source material (e.g., crude oils from different reservoirs, different regions of origin for plant extract material) can be expected to result in variability in either the presence or relative percentage of substance constituents. Although it is recognized that UVCBs can be variable, there is little information on the extent of variability for most products.

## DEVELOPMENT OF A TIERED SUBSTANCE IDENTIFICATION FRAMEWORK

As noted earlier, the challenge of risk assessment is often to determine whether the available data on a substance's composition are sufficient to conduct a reliable and robust evaluation. A reliable risk assessment collects sufficient exposure and hazard data to allow enough precision for risk‐management decisions. For example, in practice, under the Canadian Environmental Protection Act of 1999, an assessment is defined as “a scientific evaluation of a chemical substance to determine the potential harm or danger it can cause to human health and/or the environment, [and] the ways in which it can happen. This allows the federal government to identify the control measures needed to avoid or prevent the potential harm” (Government of Canada, 1999).

The characterization of UVCBs provides information that allows people to determine whether the substance is considered safe for its intended use. Or, put another way, do the available data decrease the uncertainty associated with the UVCB? Product information and process information often provide useful compositional data and help direct follow‐up analyses to reduce specific areas of uncertainty. For most UVCBs, starting with process information is the most basic and sensible thing to do.

Usually, product quality assurance/quality control (QA/QC) requires analysis of major constituents and describes both the synthetic or extraction processes and the qualities of the starting materials (including known contaminants). Available gas chromatography (GC) and liquid chromatography data can provide estimates of physical–chemical properties (e.g., log octanol–water partitioning coefficient [*K*_OW_], boiling point) that can be used to model unidentified substances for hazards. Specific analytical detectors can determine the presence or absence of functional groups or elements of concern, such as halides.

**Figure 1:**
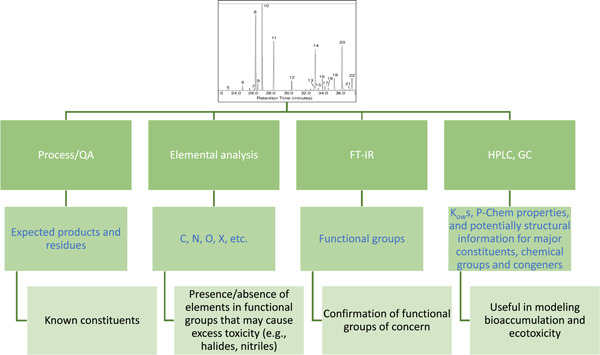
Example of a Tier 0 analytical scheme. QA = quality assurance; FT‐IR = Fourier‐transform infrared spectroscopy; HPLC = high‐performance liquid chromatography; GC = gas chromatography; *K*_OW_ = octanol–water partitioning coefficient; P‐Chem = physicochemical.

After the discussion in the workshop on the kinds of compositional information that may be available via process knowledge and QA/QC, a scheme was developed to identify the different analytical sources of information that can be developed prior to a more robust, and often higher‐tier, GC mass spectrometry (GC‐MS; or GC‐MS/MS, etc.) substance characterization (Figure 1).

### Rationale for a tiered approach

**Figure 2:**
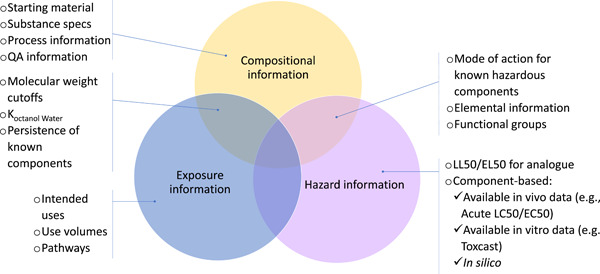
Interdependence of Tier 0 compositional, exposure, and hazard information. QA = quality assurance; *K*_Octanol Water_ = octanol–water partitioning coefficient; LL50 = median lethal limit; EL50 = median effective limit; LC50 = median lethal concentration; EC50 = median effective concentration.

Evaluating the risks associated with UVCBs requires compositional, exposure, and hazard information about the substances of interest and their constituents. These three categories of information and their associated uncertainties are not entirely independent of one another. Knowledge of substance composition will also produce information on the hazards inherent in a substance's constituents or groups of constituents, as well as their fate and transport in various environmental compartments. Figure 2 shows the potential for overlap in compositional, exposure, and hazard data. Better substance characterization tends to decrease uncertainty about substance composition and facilitate hazard and exposure characterization. This could explain why full substance characterization may often seem like the obvious answer to a robust risk assessment. However, the chemical complexity of UVCBs can make the complete elucidation of their composition difficult and may not always be justified, in terms of time and resources, to ensure that the risk evaluation will be protective of human health and the environment.

In some cases, available substance characterization, such as the identification of major generic structures, could provide valuable information for risk characterization, such as the presence of potentially persistent elements or moieties. This information could be combined with complementary exposure data, such as production and use volumes, use scenarios, and discharge volumes, to inform the magnitude of the risk of the “whole substance” to the environment. Consequently, one could argue that a tiered collection of exposure, characterization, and hazard data, starting with coarse information (Tier 0) and progressing toward increasingly refined and complex data that require more involved analytical techniques and resource mobilization, could help make the risk assessment of UVCBs more fit‐for‐purpose. With this approach, substance characterization strategies would be tailored to collect only the necessary data to make reliable and conservative risk‐based decisions and ultimately ensure a more efficient use of time and resources.

### Tiered substance characterization

During the 2018 workshop, participants focused on delineating a tiered approach to substance characterization only. The integration of the exposure and hazard components will be addressed later, and the complete approach will be the subject of a future publication. Particular attention was paid to the definition of “Tier 0” characterization information. Such information includes substance specifications, production process knowledge (e.g., starting materials, intermediates, and by‐products), as well as data provided by a basic set of characterization tests (Figure 1). Tier 0 also provides crude exposure and hazard data. Information on QA/QC will confirm that the substance meets the desired technical specifications and may reveal the presence of unexpected unknown constituents.

Basic hazard information will be derived from analytical techniques like Fourier‐transform infrared spectroscopy, which could help identify moieties of potential environmental concern, such as aromatic structures and reactive amines, as well as elemental analyses which, by revealing the presence of halogen atoms, heavy metals, and inorganics, could suggest the possible environmental persistence of certain constituents, even though their precise chemical structures may not be known at the Tier 0 stage. Finally, simple chromatographic techniques, which provide retention times and *K*_OW_s, could produce information on whether the constituents may be grouped and may persist and bioaccumulate in the environment.

For example, substance constituents with log *K*_OW_ values (as determined by OECD guideline 117 [2022]) >4.5 (that the European Union determined is the bioaccumulation screening criterion for persistent, bioaccumulative, and toxic and very persistent and very bioaccumulative assessments [ECHA, 2017a]) may indicate a significant bioaccumulation potential for these constituents and will require further investigation. In cases where hazard flags (e.g., high *K*_OW_s or the presence of halogens, aromatic structures, or phosphate moieties) indicate the presence of potentially persistent, bioaccumulative, and/or toxic constituents, more thorough characterization may be warranted, depending on the magnitude of exposure, to elucidate the structure of potential risk drivers.

**Figure 3:**
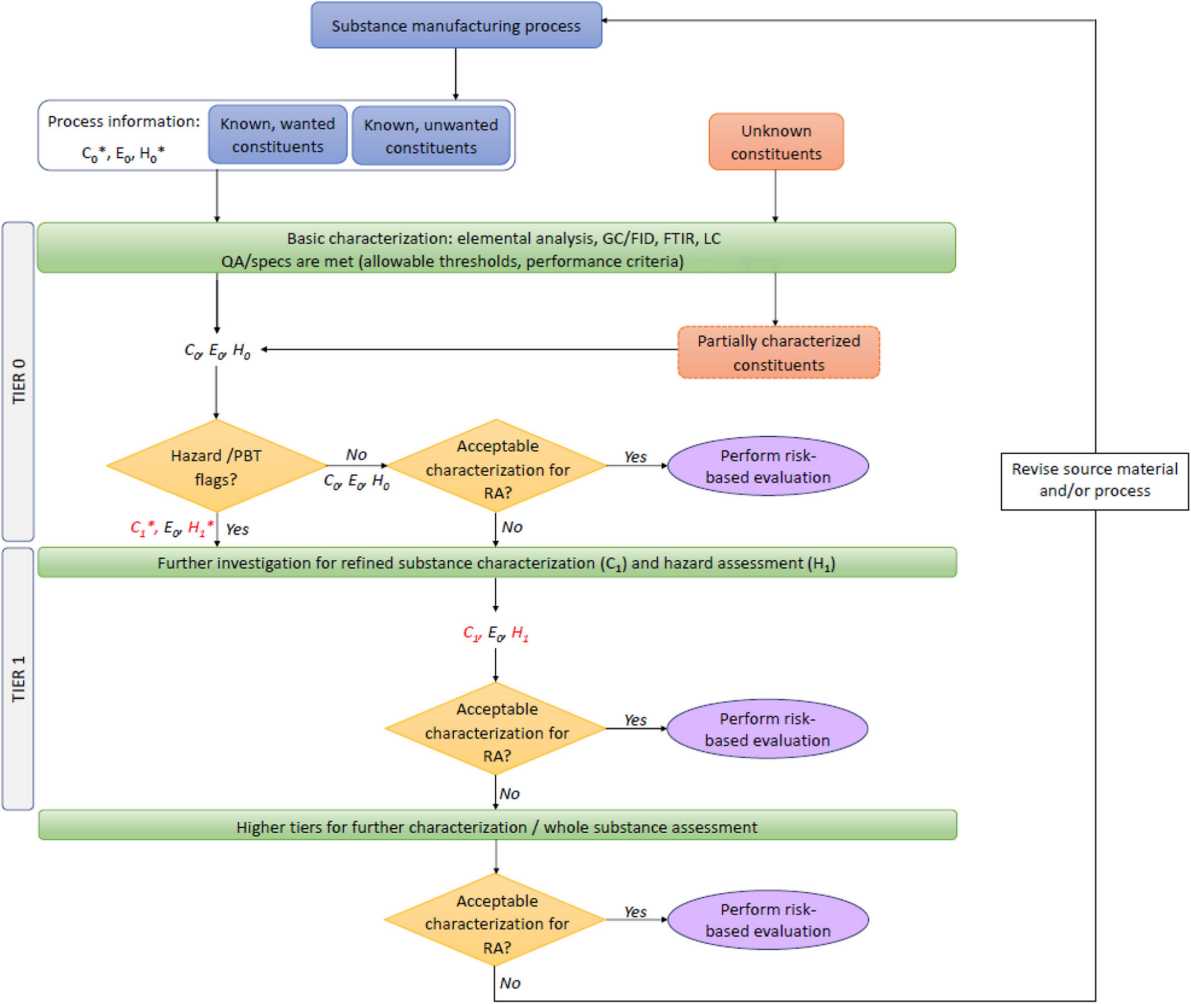
Tiered unknown or variable composition, complex reaction products, and biological material (UVCB) characterization scheme for UVCBs, where *C*_*x*_, *E*_*x*_, and *H*_*x*_ represent Tier *x* characterization, exposure, and hazard, respectively, with *x* = 0 or 1. Asterisks designate data that have not yet been confirmed via testing. GC/FID = gas chromatography/flame ionization detection; FTIR = Fourier‐transform infrared spectroscopy; LC = liquid chromatography; QA = quality assurance; PBT = persistent, bioaccumulative, and toxic; RA = risk assessment.

Figure 3 is a generic illustration of how Tier 0 characterization information can be used in the first stage of risk assessment and how the result of the Tier 0 assessment (involving Tier 0 exposure information not shown) will determine whether the substance should undergo further characterization with a Tier 1 analysis, which will not be discussed in the present study. The Tier 0 characterization stage provides some valuable exposure and hazard information—a scientific evaluation of a chemical substance to determine the potential harm or danger it can cause to human health and/or the environment, and the ways in which it can happen. This allows the federal government to identify the control measures needed to avoid or prevent the potential harm.

### In silico or predictive approaches

A complementary methodology can include in silico predictive tools. This approach will depend on the results of the Tier 0 analytical scheme.

When empirical information is not available or adequate for the characterization of a UVCB for assessment, a predictive approach may be taken to generate a list of all possible constituents present. With some experience in industrial organic chemistry and synthetic organic chemistry, one may be able to reliably predict these possible constituents' chemical structures based on nomenclature and a few details on the reaction scheme (e.g., reactant ratios, catalysts used, temperature, and purification steps), along with the source of plant or animal material and/or the type of extraction.

Ultimately, the procedure for enumerating all of the possible constituents in a complex UVCB may depend on some level of automation via Markush enumeration, a chemical structural depiction that lists classes or groups of chemical structures around a common a fixed scaffold or core structure. It is available in several software products (see MarvinSketch, 2017). Any known physical–chemical parameters for the whole UVCB may then be used to narrow down the list of all enumerated structures to only those that agree with the chemical space defined by whole‐substance data. The method described by Dimitrov et al. (2015) also relies on this type of prediction and enumeration of possible components.

The main disadvantage of an enumeration approach is that a large number of possible components may be generated that require a large amount of computational power to refine, and there has been little guidance on how to proceed. Also, the results of these approaches will not yield proportion values for chemical subclasses or components. This outcome could lead to a very conservative representation of a substance that would likely need to be verified with empirical data available from the open literature or industrial stakeholders.

## DISCUSSION

### Regulatory challenges when using a Tier 0 scheme

Most regulatory agencies employ a weight‐of‐evidence (WoE) approach to determine risk, which uses all relevant hazard and exposure information for a substance (Government of Canada, 2017; OECD, 2019). The WoE approach is most conclusive and powerful when multiple independent lines of evidence (e.g., bioaccumulation potential, toxicity threshold, exposure concentration) are available to make the risk determination. In early tiers of risk assessment, the information available for many substances is often scarce; and in the case of UVCBs, it tends to be based on limited data.

Making decisions based on Tier 0 information puts a lot of pressure on the accuracy and relevance of this limited information. Increasing the number of independent lines of evidence that support a particular decision in the decision tree shown in Figure 3 would improve the process and minimize the possibility of having a false‐negative conclusion. It is equally important to balance time and resources in regulatory assessment to ensure that regulators spend the most amount of time characterizing and risk‐assessing those substances that are most problematic for the environment and human health and avoid spending time on false positives (substances that are screened in for assessment because of some hazard or risk flag but are deemed to be not of concern in higher‐tier risk assessments).

Regulators are often responsible for the prioritization of large numbers of substances. (Environmental and Climate Change Canada, the Canadian Domestic Substance List [2019] contains more than 28 000 chemicals.) The collection of base‐level information will assist in this task, but collecting these data often requires a mandatory survey of chemical substance manufacturers and users, especially if a substance is only imported into a country and not manufactured there. Reaching out to respondents to request these often technical data and ensuring that they can be provided is an enormous task. It is possible that supply‐chain knowledge may be limited, and there could be many gaps in the Tier 0 information provided to regulators. In addition, if the mandatory survey asks respondents to generate new data, it may be up to the regulator to provide methodologies and ensure that well‐standardized approaches using readily available tools and equipment are employed. Lastly, regulators require internal data quality verification and validation techniques to ensure that the data collected are accurate and informative.

### Risk‐assessment challenges

Despite the challenges of assessing UVCBs, it is essential that their risk assessment be protective of the environment. The tendency is toward full‐substance characterization to shed light on every single substance constituent, then to single out the ones that could be hazardous. Although this approach aims to minimize uncertainty, it overlooks exposure information, including information provided by basic substance characterization (Tier 0) data.

Therefore, it seems worthwhile to examine current risk‐assessment practices and ask whether they accurately inform risk evaluation and whether they are well adapted to all UVCBs. Could they potentially be improved, for example, by integrating multiple lines of evidence that include hazard and exposure data and by being better tailored to different substances or substance categories and uses?

The partial framework described in the present study is an attempt to address this question and present UVCB risk assessment in a different light. It is meant to initiate a conversation among stakeholders on how to develop an effective and practical way to characterize and risk‐assess UVCBs. It illustrates how the focus could be shifted from full‐substance characterization for hazard identification to a more holistic approach that considers what data are needed to accurately evaluate risks.

Like all new concepts, this one is bound to be refined over time. It will benefit from the input of various experts in the field, who will help build confidence in the process. Two case studies on kerosene and rosin glycerol esters, which were developed to ground‐truth the proposed framework, are presented as Supporting Information.

## CONCLUSIONS

The approach described in the present study is the first part of a broader risk‐assessment framework, which will be further developed in future publications. Tier 0 exposure and hazard information provided by the Tier 0 characterization step (Figure 2) can be combined with additional basic exposure information, such as production and use volumes, to conduct a Tier 0 assessment of a substance. If a Tier 0 assessment can determine with confidence that the risk associated with the substance of interest is acceptable, no additional characterization data will need to be generated. However, should the degree of uncertainty of the risk assessment (including substance composition, exposure assessment, and hazard evaluation) be unacceptable, either the exposure evaluation should be refined (e.g., by accounting for partial removal of the substance in wastewater‐treatment plants) or the substance should undergo further characterization in a Tier 1 assessment.

If the risk assessment is still not satisfactory, it may be necessary to include higher‐tier exposure information, such as biodegradation data or environmental measurements. It should be noted that, although Tier 0 uses coarse and readily available information (Figure 2), the definition of higher characterization tiers may depend on whether exposure and hazard information is sufficient to reduce the uncertainty of the risk assessment so that it can be used by decision makers. Deciding to move to higher characterization tiers will often have to be determined on a case‐by‐case basis. Whether or not information on substance end use produces more precise risk evaluations will be further developed in future work integrating the exposure component to the present framework.

Even though Tier 0 characterization data may seem limited because they only partially inform substance composition, they may still reduce uncertainty in substance characterization sufficiently to make risk‐based decisions and rule out the need for more elaborate substance characterization and testing. This would be especially true if the substance is devoid of hazardous constituents and will not persist in the environment. It is important to note that a Tier 0 assessment is a conservative screening step, and if it produces any uncertainty or a hazard or persistence flag, the matter should be further investigated by integrating higher‐tier information into the assessment. Tier 0 exposure and hazard information must be carefully considered to ensure that the risk assessment is not only robust but also fit‐for‐purpose and that resources are not spent on unnecessary characterization efforts.

The HESI UVCB Committee is currently working on how to integrate exposure information into the framework featured in the present study.

## Supporting Information

The Supporting Information is available on the Wiley Online Library at https:/10.1002/etc.5462.

## Disclaimer

The authors declare no conflict of interest. The views expressed in the present study are those of the authors and do not represent the views of their respective organizations.

## Author Contributions Statement

**Daniel Salvito, Marc Fernandez**: Conceptualization; Supervision; Writing—original draft; Writing—review. **Sandrine E. Déglin**: Conceptualization; Project administration; Supervision; Writing—original draft; Writing—review. **Jeremey S. Arey, Matthew MacLeod**: Conceptualization; Writing—review. **Delina Y. Lyon, Nelson Lawson**: Conceptualization; Writing—original draft; Writing—review.

## Supporting information

This article contains online‐only Supporting Information.

Supporting information.

Supporting information.

## Data Availability

The authors are not reporting any experimental or data collection work.
