# Supplementary material for: The Path to UVCB Ecological Risk Assessment: Grappling with Substance Characterization
Source: Environ Toxicol Chem. 2022 Sep 30;41(11):2649–57. doi: 10.1002/etc.5462 (PMC9828001; doi:10.1002/etc.5462)
Supplement: Supplementary file 1 — Supporting information. [file ETC-41-2649-s002.docx]

**CASE STUDY #2 KEROSENE**

**KEROSENE SOURCE MATERIAL**

**Origins and Production Process**

Petroleum substances are examples of UVCBs that are extracted or purified from natural sources. Crude oil is an extremely complex type of hydrocarbon substance, the exact composition and properties of which will vary by source. Crude oils can be classified by constituents or physicochemical property, like gravity or boiling point. Depending on the desired product, the refining and manufacturing processes can take a crude oil that contains thousands of different hydrocarbon molecules to a final product that has a few types of hydrocarbons or even a relatively pure mono-constituent product. A typical crude oil refinery uses a combination of distilling (that separates molecules by boiling point, with higher molecular weight constituents having a higher boiling point [Figure 1]), cracking (generating smaller hydrocarbon molecules by breaking up bigger ones), reforming (catalyzing molecular rearrangements), treating (removing contaminants like sulfur), and blending to achieve products that achieve the desired specifications.

Defining the composition of petroleum-derived substances can therefore be fairly straightforward for relatively pure substances or extremely complex substances. Kerosene jet fuel is an example of a petroleum substance which is fairly complex but for which there is a substantial amount of compositional information. As defined by the Petroleum High Production Volume Testing Group and Concawe, kerosene or kerosine is the generic name for the lighter end of middle distillates, consisting predominantly of hydrocarbons in the C9-C16 carbon number range with a boiling range of approximately 302 to 554 °F (150 to 290 °C)(Figure 1)(API 2010, CONCAWE 1999). It is commonly used in jet fuel and for heating/cooking.


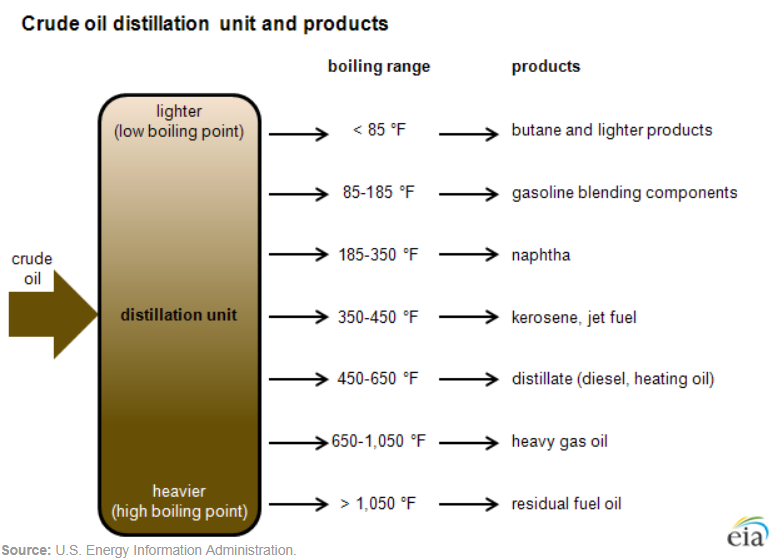


**Figure 1 – Generic crude oil refining process (Source: EIA. https://www.eia.gov/energyexplained/oil-and-petroleum-products/refining-crude-oil-the-refining-process.php)**

**ANALYSIS OF THE PRODUCT FOR TIER 0 CHARACTERIZATION**

There are several substances with different CAS numbers that lie within the kerosene range. Depending on the specific application, different properties such as the boiling point range or the aromatic hydrocarbon content can be adjusted, which shifts the composition of the specific kerosene.

Following the tiered approach laid out in this paper, Tier 0 information would include a description of the product (technical specifications) and information on the manufacturing/refining process, as well as readily available compositional information.

TIER (0) HAZARD AND EXPOSURE INFORMATION

Tier 0 composition information on kerosene will provide some indication on the substance hazard and fate (hence, exposure). From a human health perspective, the aromatic content would be the risk driving factor. For environmental hazards, it is possible to use *in silico* predictions to estimate the range of ecotoxicity associated with the carbon number range. It is also possible to predict the range of expected biodegradability, again using predictive models like BioHCWin. However, these types of estimations tend to give a very large range of results, since the estimations are made on individual constituents that span the range of the substance. A highly conservative assessment would be to take the worst-case values for constituents, meaning the greatest toxicity, longest biodegradation half-life, etc. However, this would not be indicative of the properties of the whole substance. Another option would be a more specific predictive model, like PetroTox, as discussed in the section below.

As with a Tier 0 hazard assessment, a Tier 0 exposure estimation would rely on large ranges of estimated values for water solubility, log K_ow_, etc. In general, due to the low water solubility of kerosene, it would be expected that it would not partition to water. Additionally, volatile constituents are expected. Probably the most significant information from a Tier 0 assessment standpoint would be the volumes and uses, which inform the exposure component of the risk assessment. The large volume alone would trigger a need for more in-depth risk assessment.

**HIGHER TIER ANALYSIS OF KEROSINE**

As petroleum substances have been used for a long time and at high volumes, there is a large amount of data available as well as new data still being generated on substance composition. Additionally, to predict aquatic toxicity of petroleum UVCBs, Concawe has developed PetroTox (<https://www.concawe.eu/reach/petrotox/>) (Redman et al. 2012). It is based on the hydrocarbon block method (HCBM) which uses analytical data, specifically GCxGC data, to group similar constituents into “hydrocarbon blocks”, and then predict the ecotoxicity of the whole substance based on the sum of the blocks (Redman et al. 2017). The toxicity is predicted based on the Target Lipid Model, which relates log(Kow) to log (LC50) for hydrocarbons with a narcotic mode of action (McGrath and di Toro, 2009).

While GCxGC analysis may be beyond a Tier 0 scope, the composition of a generic kerosene was described as follows:


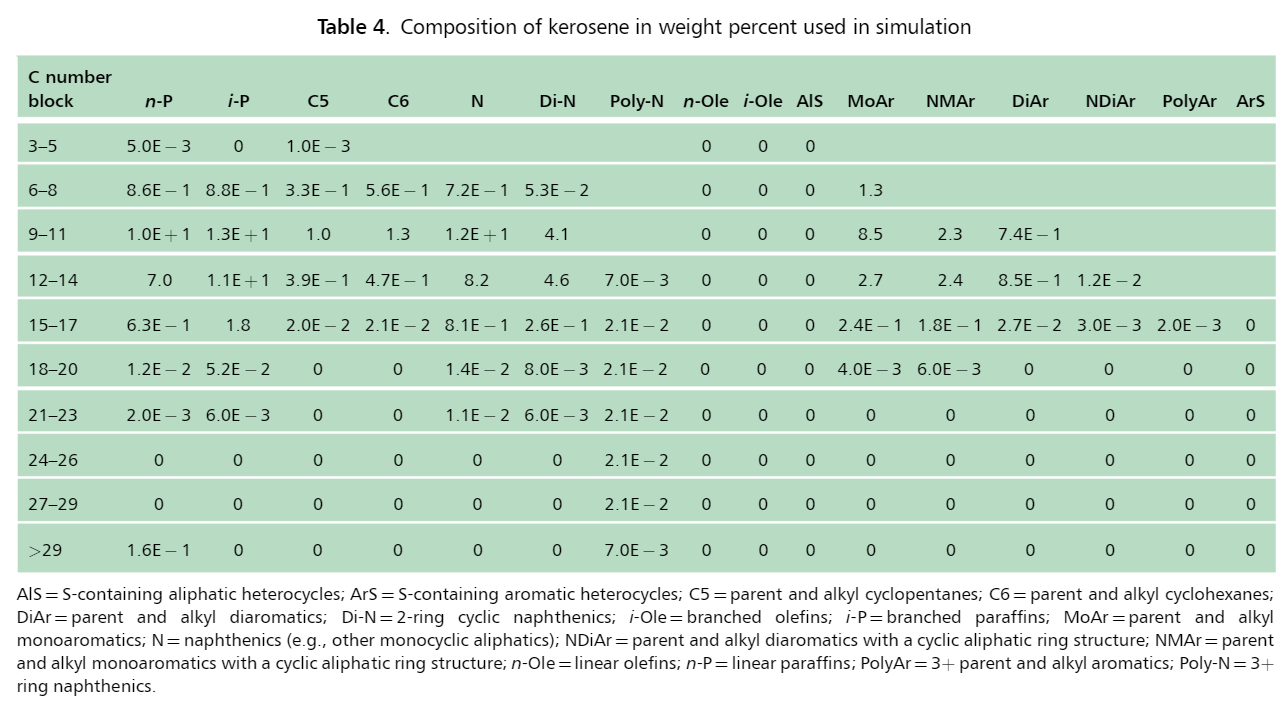


This hydrocarbon block information can be fed into the PetroTox model to predict toxicity to environmental systems (Redman et al. 2014).

**References**

API. 2010. Kerosene/Jet fuel category assessment document. Available at: <https://petroleumhpv.org/petroleum-substances-and-categories/~/media/37A083A569294403AD230CB504AB17A6.ashx>

CONCAWE. 1999. Exposure profile: Kerosines/Jet fuels. Available at: <https://www.concawe.eu/wp-content/uploads/2017/01/2002-00218-01-e.pdf>

McGrath JA and Di Toro DM. 2009. Validation of the target lipid model for toxicity assessment of residual petroleum constituents: monocyclic and polycyclic aromatic hydrocarbons Environ Toxicol Chem 28(6):1130-48

Redman AD, Parkerton TF, McGrath JA, Di Toro DM. 2012. PETROTOX: an aquatic toxicity model for petroleum substances. *Environ Toxicol Chem* 31:2498–2506.

Redman AD, Parkerton TF, Comber MHI, Paumen ML, Eadsforth CV, Dmytrasz B, King D, Warren CS, den Haan K, Djemel N. 2014. PETRORISK: a risk assessment framework for petroleum substances. *Integr Environ Assess Manage* 10:437–448.

Redman AD, Parkerton TF, Paumen ML, Butelr JD, Letinski DJ, den Haan K. 2017. A re‐evaluation of PETROTOX for predicting acute and chronic toxicity of petroleum substances**.** *Environ Toxicol Chem* 36(8):2254-2242
