# Supplementary material for: The Path to UVCB Ecological Risk Assessment: Grappling with Substance Characterization
Source: Environ Toxicol Chem. 2022 Sep 30;41(11):2649–57. doi: 10.1002/etc.5462 (PMC9828001; doi:10.1002/etc.5462)
Supplement: Supplementary file 2 — Supporting information. [file ETC-41-2649-s001.docx]

**CASE STUDY #1 GLYCEROL ESTER OF ROSIN**

The objective of this case study is to demonstrate how a Tier 0 risk assessment works on a UVCB substance – the glycerol ester of rosin (GER). As stated earlier in this paper, the idea behind a Tier 0 risk assessment is to determine – from knowledge of the major constituents, the starting materials, knowledge of the process by which the substance is made, and relatively simple analytical work such as high pressure liquid chromatography (HPLC), gas-liquid chromatography (GLC), Fourier-transfer infrared spectroscopy (FTIR), functional group analyses, impurities, estimates of molecular weight, and other readily available published information – whether a given substance is likely or unlikely to be of concern in a formal risk assessment, or whether further tests and studies are necessary to reach a reasonable conclusion.

**ROSINS**

***Origins and production processes***

Rosin is a solid form of resin obtained from conifers (mainly pines) and some other plants. It chiefly consists of various diterpene resin acids. Gum rosin is produced by tapping live pine trees to make a liquid oleoresin. The turpentine portion is distilled away leaving a solid resin. Wood rosin is produced by extracting chipped old pine stumps with a hydrocarbon solvent, removing the colored impurities by liquid-extraction with another solvent such as furfural, followed by solvent evaporation. Tall oil rosin is produced as a byproduct of the kraft pulping of conifers. In this process, lignin, hemicellulose, rosin, and fatty acids are removed from the cellulose by heating with an alkaline solution of sodium hydroxide and sodium sulfide. The resulting black liquor is concentrated for burning in a recovery boiler and the rosin and fatty acid soaps float to the top, are skimmed off, acidified, and distilled to form tall oil rosin and tall oil fatty acids.

***Composition***

The resin acids found in rosins are mainly of two skeletal types:


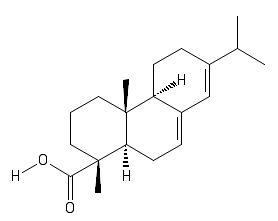


Abietic Acid


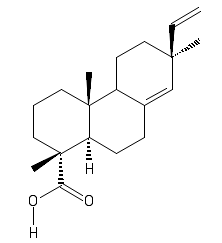


Pimaric Acid

The abietic-type acids consist of varying percentages of resin acids. Abietic, palustric, and neoabietic acids have two double bonds, while dihydroabietic acids have only one, and dehydroabietic acid has an aromatic ring.

The pimaric acids consist of varying percentages of resin acids with two double bonds, such as pimaric, isopimaric and sandaracopimaric acids. Some dihydropimaric acids are also present. Depending upon the source (mainly tall oil rosin) there may be minor concentrations of dimerized rosin and fatty acids. The composition of rosins varies, depending upon tree species, location, and mode of processing. Rosins also contain varying concentrations of neutral substances.

**TYPICAL COMPOSITION OF THE COMMON RESIN ACIDS IN SOME US ROSINS^[[1]](#footnote-1)^**

| **RESIN ACID** | **TALL OIL** | **WOOD** | **GUM** |
| --- | --- | --- | --- |
| Pimaric | 4.4 | 7.1 | 4.5 |
| Sandaracopimaric | 3.9 | 2.0 | 1.3 |
| Communic | 1.0 | - | - |
| Levopimaric | - | - | 1.8 |
| Palustric | 8.2 | 8.2 | 21.2 |
| Isopimaric | 11.4 | 15.5 | 17.4 |
| Abietic | 37.8 | 50.8 | 23.7 |
| Dehydroabietic | 18.2 | 7.9 | 5.3 |
| Neoabietic | 3.3 | 4.7 | 19.1 |

Although these three rosins can come from the same pine tree source, they can differ in composition and in impurities that affect their uses. For example, both gum and wood rosins can be hydrogenated to saturate the double bonds and improve oxidative stability. Partial hydrogenation is accomplished over a nickel catalyst at lower pressures, but full hydrogenation requires higher pressures or the presence of a precious metal catalyst. Tall oil rosin contains too much sulfur impurities to make hydrogenation economical.

**GLYCEROL ESTER OF ROSIN**

Many different derivatives can be made from rosin; one of the most important is the *glycerol ester*. (GER), sometimes called “ester gum.” Its Chemical Abstracts number is 8050-31-5.

***Composition***

The main constituents of GER by HPLC are the triester, the diester, the monoester, unreacted rosin and neutrals, and possibly other minor impurities. A typical analysis would be:

| **SPECIES** | **CONCENTRATION** |
| --- | --- |
| Tri and diester | 82% |
| Monoester | 2.2 |
| Neutrals | 13.2 |
| Resin acids | 2.6 |

The table below lists the average compositions of three samples each the glycerol ester (GE) of tall oil rosin and wood rosin from the European Food Safety Agency (EFSA).^[[2]](#footnote-2)^ They are compared to the compositions of glycerol esters of partially and fully hydrogenated wood rosin. It is likely that the differences in the proportion of mono-, di- and triesters are due more to the stoichiometry and processing conditions rather than to the inherent differences between the two rosins. By increasing the ratio of glycerol to rosin, the free resin acids will decrease and the monoesters will increase relative to the di- and triesters. Decreasing the glycerol will increase the triesters. Severe vacuum stripping will reduce the concentration of free resin acids and neutrals. The end-use will determine the optimum concentrations, but under most conditions, a high concentration of tri- and diesters and a low concentration of free resin acids and neutrals are desirable. Thus, a good analysis of the GER is needed before a complete hazard analysis can be done.

| **SPECIES** | **TALL OIL ROSIN**  **(%)** | **WOOD ROSIN**  **(%)** | **PARTIALLY HYDROGENATED WOOD ROSIN (%)** | **FULLY HYDROGENATED WOOD ROSIN (%)** |
| --- | --- | --- | --- | --- |
| Resin acids | 3.8 | 4.5 | 4.4 | 5.2 |
| Monoesters | 12.7 | 13.0 | 3.8 | 2.4 |
| Diesters | 63.3 | 73.0 | 21.9 | 19.5 |
| Triesters | 20.3 | 9.7 | 57.6 | 66.3 |

Gaefvert et al.^[[3]](#footnote-3)^ showed that the following esters were present when only abietic acid was the starting material:


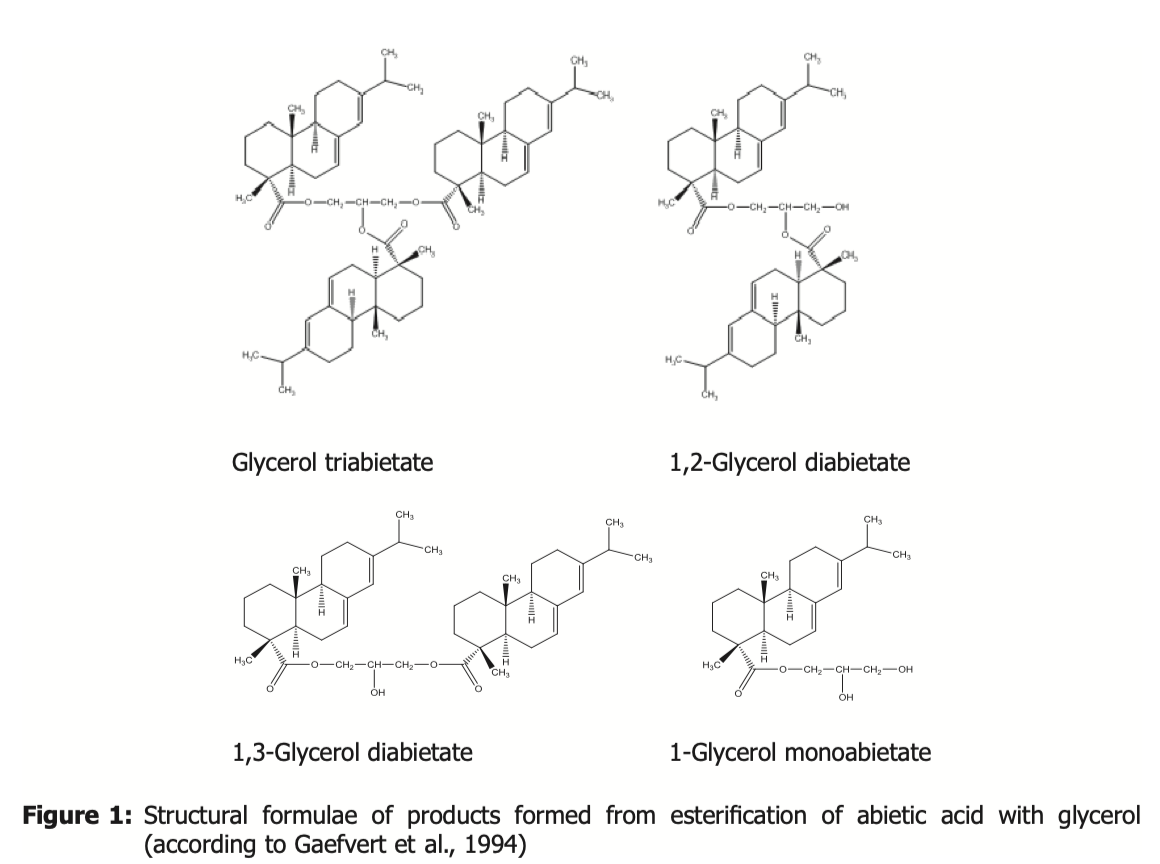


When rosin is the starting material, every resin acid can form all of these esters. Most likely the composition of the resin acids in the esters reflects the composition of the starting rosin; therefore, the number of individual chemical species in GER is very large and it would be impractical (and unnecessary) to try to deal with each individually.

GER is prepared from all three types of starting rosins, depending upon the desired end use. For example, the glycerol ester of wood rosin is used primarily in high-value end-uses where it has regulatory approval, such as emulsifiers in fruit drinks and spirits, citrus coatings, chewing gum, or in cosmetic or personal care products. The glycerol ester of gum rosin is approved in some (but not all) countries for the same uses. The glycerol ester of tall oil rosin is usually of lower value due to the residual odor of low levels of sulfur or phenolic impurities from the kraft pulping process, and is used mainly in adhesives. It is not approved for food uses in most countries. Tall oil rosin also contains minor concentrations of fatty acids that preferentially react with the glycerol, confusing the situation further.

The presence of neutrals in GER from gum and wood rosins generally fall into the following classes: methyl esters of rosin, resin hydrocarbons, diterpene aldehydes, and diterpene alcohols and noralcohols based on the composition of the starting wood rosin.^[[4]](#footnote-4)^ The neutrals from all oil rosin contains mostly resin alcohols and sterols such as beta-sitosterol.^[[5]](#footnote-5)^ Diterpene aldehydes have excited significant interest by regulatory authorities due to QSAR models that purport to show that they may be an environmental hazard. These models are somewhat suspect because the learning data set does not include similar tertiary aldehydes.

***Production process***

Molten rosin is charged under inert gas in a batch reactor equipped with an agitator, reflux condenser, and vacuum system, followed by the addition of a catalyst (if any). A variety of acid or base reaction catalysts can be used as well as metal salts, such as calcium acetate. Often, no catalyst is used, depending on the end-use application. A stoichiometric quantity of glycerol is then charged to achieve the maximum concentration of triesters in the GER. The reaction mixture is heated at a standard ramp rate to the desired top temperature, typically in the 240-280°C range. The product is sampled until an initial acid number target is reached, at which point vacuum is applied and steam distillation or stripping is used to remove rosin oils (unreacted resin acids, neutrals and any decarboxylated rosin). Generally, it is desirable to strip out as much unreacted resin acids and neutrals as is economically possible. When the product is on-grade for acid number, softening point, and other properties, the resin is cooled and packaged, usually in flake or pastille form.

Depending on the feed rosin type and the end use application, procedural modifications such as the use of disproportionation catalysts such as nonyl phenol sulfide, color improvement additives such as hypophosphorus acid, and/or antioxidants (generally phenolic types) may be made.

**ANALYSIS OF THE PRODUCT FOR TIER 0 CHARACTERIZATION**

- The characteristics of the final product can be determined by high pressure liquid chromatography, which results in the separation of the following categories of chemicals, by size:Glycerol esters: triesters (MW ~ 945), diesters (MW ~ 661), and monoesters (MW ~377). In general the reaction stoichiometry is chosen to maximize the di- and triester content.
- Unreacted resin acids (usually kept to a minimum to maintain the softening point).
- Neutrals.
- Fatty esters would be present only if tall oil rosin were the feedstock.

Other tests or knowledge of the process and starting materials can identify:

- The unreacted resin acids and the neutrals, by gas-liquid chromatography (GLC).
- The presence of minor impurities such as phenolics, or color lightening additives such as phenolic residues, by FTIR.
- The presence of metals, sulfur compounds if tall oil rosin is the feedstock, or phosphorus, if a disproportionating catalyst was used, by elemental analyses.^[[6]](#footnote-6)^

**TIER 0 HAZARD AND EXPOSURE INFORMATION**

***Hazard (H_0_)***

The different groups identified by chromatography can be associated with different modes of toxic action (MoA) categories. For example, according to the Verhaar scheme, esters are expected to belong to a Class 2 MoA, characterized by polar narcosis. In particular, the tri- and diesters are, in general, too large to interact with living systems. The identification of unusual functional groups by FTIR or elements by analysis, after the Tier 0 compositional analysis, would require the compilation of Tier 1 hazard information.

Basic hazard data can often be found by using the USEPA CompTox database. Unavailable data can often be calculated using QSAR techniques in CompTox. The ECHA REACH dossier database can also be helpful. It is apparent that the glycerol esters of rosin are, in general, innocuous to humans and to the environment.

Sometimes a risk assessment is publicly available, for example, from the Canadian government - Environment & Climate Change Canada (ECCC) and Health Canada (HC). A Final Risk Assessment for hydrogenated rosin, glycerol ester was published by ECCC and HC and many of the data can be read across to GER for a Tier 0 risk assessment.^[[7]](#footnote-7)^ A Draft Screening Risk Assessment of Resins and Rosins was published by the ECCC and HC, but it is only in draft form and many contrary comments were submitted from industry.^[[8]](#footnote-8)^ Moreover, although it evaluates the hazards and exposure to Canadians and the Canadian environment; only rosin, dehydroabietic acid, rosin and hydrogenated rosin, and methyl esters were evaluated. GER was not evaluated. Still, the data and estimations from such studies may be useful in a Tier 0 risk assessment since unreacted resin acids are present in GER.

The European Food Safety Agency (EFSA) published human health risk assessments for GER made from wood rosin^[[9]](#footnote-9)^ and from tall oil rosin^[[10]](#footnote-10)^ containing much useful compositional and human health data.

To summarize, the glycerol tri- and di-esters are generally considered to be too stable and too large to interact with living systems.^6^ Only the monoester is thought to be a possible environmental or human health hazard, by itself or possibly due to hydrolysis. From the known chemistry of rosin esters, the glycerol esters would be expected to be fairly stable to hydrolysis due to their hindered tertiary carboxylic ester structure. In any case, the *in vitro* hydrolysis end-products would be glycerol and the resin acids from the starting rosin.

In most cases, the resin acid composition in the esters would be similar to that of the starting rosin, unless the rosin were subjected to a disproportionation step during the heat-up phase of the esterification. In such a case, the concentration of the aromatic dehydroabietic acid and the dihydro-acids would be significantly higher, and the abietic-type acids significantly lower. The actual composition of the esterified resin acids can be determined by reducing the ester using lithium aluminum hydride or other strong reducing agents, followed by GLC of the resin alcohols, but this would be a higher order, Tier 1, hazard assessment.

More recently, ECHA calculated that the glycerol monoester of hydrogenated rosin might be a Persistent, Bioaccumulative, and Toxic (PBT) substance in Europe, thus making the entire glycerol ester of hydrogenated rosin a PBT substance subject to authorization, despite its minor concentration. An industry group prepared a relatively pure sample of the monoester and tested it for biodegradability, finding that it was indeed not persistent and therefore not a PBT.^[[11]](#footnote-11)^

Since glycerol is not hazardous or toxic, the unreacted resin acids and any minor amounts of resin from hydrolysis, plus the neutrals, would seem to be the main sources of any possible hazardous substances in the glycerol ester of rosin. The environmental effects of individual resin acids in the GER would be expected to be small, since their total concentration in GER is small. They have also been evaluated in several studies summarized in Ref. 5. The effects of individual resin acids on fish, daphnia, and algae are well known and are available in CompTox. Curiously, it is the isopimaric acid that seems to have the most aquatic toxicity, at least to daphnia.

The individual resin acids have not been examined in detail for human health effects (with the exception of dehydroabietic acid).^5,6,7^ QSAR calculations shown below indicate low acute toxicity. However, the studies on individual resin acids referred to in Ref. 6 indicate that neoabietic acid is a possible mutagen and its concentration in the glycerol ester of wood rosin is limited in European food applications to 0.05%. Other sources of rosin can contain different, and sometimes unusual, resin acids, so the source rosin should be carefully checked. For example, some samples of the rosin from *Pinus brutia* have been shown to contain significant concentrations of kauradienoic acid^[[12]](#footnote-12)^ a known abortifactant.^[[13]](#footnote-13)^

| **RESIN ACID** | **PREDICTED LD_50_ FOR RATS** |
| --- | --- |
| Dihydroabietic acid | 4293 |
| Abietic acid | 3538 |
| Neoabietic acid | 3505 |
| Palustric acid | 3379 |
| Pimaric acid | 2956 |
| Isopimaric acid | 2915 |
| Dihydropimaric acid | 1869 |
| Dihydroisopimaric acid | 1857 |
| Dehydroabietic acid | 1710* |
| Communic acid | 212 |
| Rosin | > 2000* |

* Measured

***Exposure (E_0_)***

Exposure data can now be obtained more easily than in the past. Most companies maintain internal marketing data on their products and product data sheets that identify end-uses. These data are publicly available, often through an Internet search. The European Chemicals Agency (ECHA) maintains qualitative and production range information on the REACH registration website.

The best data source is the United States Environmental Agency (USEPA) CompTox website where much production and use data is easily found on the more common substances. Information is often available from trade conference presentations or from general texts such as the Encyclopedia of Chemical Technology, which is unfortunately often out of date and not much use for relatively new UVCB products. The USEPA Toxic Release Inventory (TRI) dataset can be useful if the substance is considered to be toxic and is in actual production.

Such data sources indicate that GER is used in lubricants and greases, adhesives and sealants, coating products, anti-freeze products, biocides, fillers, putties, plasters, modeling clay, polishes and waxes, textiles, chewing gum base, weighting agent in citrus drinks, perfumery, and many other consumer products. Its main use is as an adhesive tackifier.

For GER, the USEPA Chemical Data Reporting database available on CompTox indicated that 8.6 M lb. was manufactured in the USA in 2016, of which 7.4 M lb. was used and the rest exported. None was imported. In Europe, between 10K and 100K metric tons were produced or imported. GER is obviously a high-volume substance, which *may* mean that a Tier 1 risk assessment is appropriate unless the hazard assessment proves to be innocuous. Unsurprisingly, it does not show up in the TRI dataset.

***Physical properties***

Often, rough exposures can be calculated using the physical properties of the GER and models of the particular end-use. These can sometimes be used to estimate the upper limits of exposure and, if sufficiently low, can result in a “no risk” scenario. This is beyond the scope of this brief case study. Measuring all the physical properties of the components of GER is not practical, and in fact few measurements have been done on the whole GER itself. A useful reference is the Final Screening Assessment for hydrogenated rosin and its esters,^5^  in which the following physical properties were estimated:

Using the EPIWIN suite of computer models, ECCC calculated the following physical properties for rosin glycerol ester:^[[14]](#footnote-14)^

Log Kow: 5.13

Water solubility by WSKOW: 0.1768

Water solubility by WATERNT: 1.075

The calculated water solubility by WSKOW compares well to that measured for the similar hydrogenated rosin glycerol ester (CAS 65997-13-9): 0.15 mg/L^[[15]](#footnote-15)^

Vapor pressure would be extremely low for rosin glycerol ester; that calculated for hydrogenated rosin glycerol ester was: glycerol mono ester, 4.09 x 10^-9^ mm. Hg; glycerol diester, 1.54 x 10^-15^; and glycerol tri ester, 4.76 x 10^-19^.^18^ This document contains other calculated physical property data for the hydrogenated rosin glycerol ester.

Calculated environmental toxicities were: Fish, acute-1.26; Daphnia, acute-0.424; Algal, acute-0.108; Algae ChV-0.089; Ready biodegradability, no, log BCF-3.252. There seems to be no probable environmental toxicity.

A great deal of human health data was presented in a recent article by EFSA pertinent to the use of rosin glycerol ester in various food products.^[[16]](#footnote-16)^ If the ester is derived from *Pinus elliottii* and *Pinus palustris* and the neoabietic acid content is kept below 0.05%, the product is generally suitable for food applications at certain low percentages in Europe and in the USA

1. *"Naval Stores"*, D.F. Zinkel and J. Russell, Editors, Pulp Chemicals Association, New York (1989), p.274. [↑](#footnote-ref-1)
2. EFSA Panel on Food Additives and Nutrient Sources added to Food (2011). Scientific Opinion on the safety of Glycerol Esters of Tall Oil Rosin for the proposed uses as a food additive. *EFSA Journal*, 9(5), 2141. Available online: www.efsa.europa.eu [↑](#footnote-ref-2)
3. Gaefvert E. et al. (1994). Allergenicity of rosin (colophony) esters (II).Glyceryl monoabietate identified as contact allergen, *Contact Dermatitis,* 31, 11-17. [↑](#footnote-ref-3)
4. EFSA ANS Panel (EFSA Panel on Food Additives and Nutrient Sources added to Food) (2018). Scientific Opinion on the re-evaluation of glycerol esters of wood rosin (E 445) as a food additive. *EFSA Journal,* 16(7), 5370. Available online: https://efsa.onlinelibrary.wiley.com/doi/full/10.2903/j.efsa.2018.5370 [↑](#footnote-ref-4)
5. EFSA Panel on Food Additives and Nutrient Sources added to Food (2011). Scientific Opinion on the safety of Glycerol Esters of Tall Oil Rosin for the proposed uses as a food additive. *EFSA Journal*, 9(5), 2141. Available online: https://efsa.onlinelibrary.wiley.com/doi/epdf/10.2903/j.efsa.2011.2141 [↑](#footnote-ref-5)
6. EFSA suggests a simple test involving heating a sample with sodium formate. Lead acetate test paper turns black in the presence of sulfur. [↑](#footnote-ref-6)
7. ECCC and HC, "Screening Assessment for the Challenge: Rosin, hydrogenated Chemical Abstracts Service Registry Number 65997-06-0; Resin acids and Rosin acids, hydrogenated, esters with pentaerythritol Chemical Abstracts Service Registry Number 64365-17-9; Resin acids and Rosin acids, hydrogenated, esters with glycerol Chemical Abstracts Service Registry Number 65997-13-9; Resin acids and Rosin acids, hydrogenated, esters with triethylene glycol Chemical Abstracts Service Registry Number 68648-53-3; January 2011." [↑](#footnote-ref-7)
8. ECCC and HC, “Draft Screening Assessment Resins and Rosins Group, June, 2019.” [↑](#footnote-ref-8)
9. EFSA ANS Panel (EFSA Panel on Food Additives and Nutrient Sources added to Food) (2018). Scientific Opinion on the re-evaluation of glycerol esters of wood rosin (E 445) as a food additive. *EFSA Journal,* 16(7), 5370. Available online: https://efsa.onlinelibrary.wiley.com/doi/full/10.2903/j.efsa.2018.5370 [↑](#footnote-ref-9)
10. EFSA Panel on Food Additives and Nutrient Sources added to Food (2011). Scientific Opinion on the safety of Glycerol Esters of Tall Oil Rosin for the proposed uses as a food additive. *EFSA Journal*, 9(5), 2141. Available online: https://efsa.onlinelibrary.wiley.com/doi/epdf/10.2903/j.efsa.2011.2141 [↑](#footnote-ref-10)
11. R.J. Guinn, “*Environmental Fate Assessment of Resin Products: The approach for EU REACh PBT Assessment of Rosin Esters,”* Presented at the ECETOC Workshop on Developing a strategy to improve the hazard and risk assessment of difficult to test multi-component substances (2016-11-02 to 04) Final result: unpublished information from the H4R Consortium. [↑](#footnote-ref-11)
12. A. Goren et al. (2010). Chemical Composition of Natural Colophony from Pinus brutia and Comparison with Synthetic Colophony. *Natural Product Communications*, 5(11), 1729. Available online: https://journals.sagepub.com/doi/pdf/10.1177/1934578X1000501105 [↑](#footnote-ref-12)
13. A. J. Gallegos, US Pat 4,491,593 (1985). [↑](#footnote-ref-13)
14. ECCC table “Green Rosin Data,” obtained from ECCC as part of the DSRA for the Resins and Rosins Grouping. [↑](#footnote-ref-14)
15. Inveresk Report 24028, data submitted to USEPA for the High Production Volume Chemicals Challenge (2004-08-13). [↑](#footnote-ref-15)
16. EFSA ANS Panel (EFSA Panel on Food Additives and Nutrient Sources added to Food) (2018). Scientific Opinion on the re-evaluation of glycerol esters of wood rosin (E 445) as a food additive. *EFSA Journal,* 16(7), 5370. Available online: https://efsa.onlinelibrary.wiley.com/doi/full/10.2903/j.efsa.2018.5370 [↑](#footnote-ref-16)
